# Polarized iridescence of the tropical carpenter bee, *Xylocopa latipes*

**DOI:** 10.1007/s00359-022-01592-9

**Published:** 2022-11-16

**Authors:** Doekele G. Stavenga, Kim Kats, Hein L. Leertouwer

**Affiliations:** 1 Groningen Institute for Evolutionary Life Sciences, University of Groningen, Nijenborgh 7, 9747AG Groningen, The Netherlands; 2 Department of Biomedical Science of Cells and Systems, University of Groningen, University Medical Center Groningen, Groningen, The Netherlands

**Keywords:** Reflectance spectrum, Structural colouration, Spectral sensitivity, Degree of polarization

## Abstract

The tropical carpenter bee, *Xylocopa latipes*, has metallic-reflecting, iridescent wings. The wing reflectance spectra for TE- and TM-polarized light depend on the angle of light incidence in a way characteristic for dielectric multilayers. Anatomy indicates the presence of melanin multilayers in the wing’s chitinous matrix. A simple optical model of melanin multilayers explains the angle dependence of the wing reflectance spectra. The wing reflections that occur upon oblique illumination exhibit colourful and strongly polarized light patterns, which may mediate intraspecific signaling and mutual recognition by conspecifics.

## Introduction

**Fig. 1:**
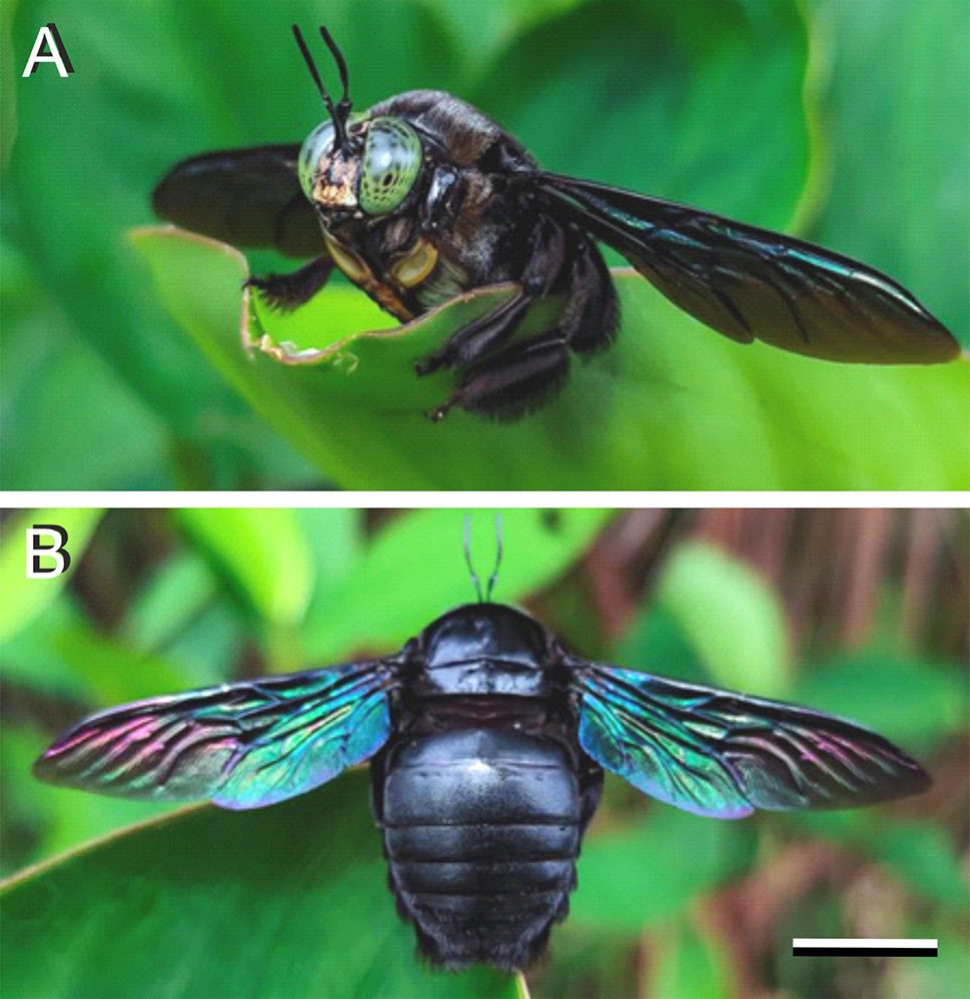
The Tropical carpenter bee, *Xylocopa latipes*. **A** Frontal view, showing the large eyes with pseudopupils. **B** Dorsal view, showing the multicoloured wings. Photographs by A. Kamaruzzaman (https://www.steemzzang.com/hive-109286/@akukamaruzzaman/xylocopa-latipes-the-tropical-carpenter-bee). Scale bar: 1 cm

Carpenter bees are species in the extensive genus *Xylocopa* that burrow into hard plant material. The tropical carpenter bee, *Xylocopa latipes*, is a particularly large, robust bee, widely dispersed throughout Southeast Asia. It is not only conspicuous due to its size, but especially because of its shiny body and metallic-reflecting, colourful wings, which vary from purple to blue or green (Fig. 1). Visual observation shows that the latter colouration is iridescent, i.e., the colours change with the angle of illumination and observation. However, observed in transmitted light, the wings have a brown to black colour. This shows that the wing colouration is structural, presumably similar to the wings of some odonates and hymenopterans, which have melanin arranged in regular layers in the wings’ chitin matrix (Vukusic et al. 2004; Sarrazin et al. 2008; Schultz and Fincke 2009; Stavenga et al. 2012; Guillermo-Ferreira et al. 2015; Guillermo-Ferreira et al. 2019; Suárez-Tovar et al. 2022). Melanin multilayers also exist in the green iridescent elytra of the Jewel beetle *Chrysochroa fulgidissima*, where an increasing angle of light incidence causes a distinct blue-shift together with a strong polarization of the reflected light (Stavenga et al. 2011).

These previous studies triggered an interest in the characteristics and optical origin of the colouration of *X. latipes*, specifically as the large eyes of *Xylocopa* carpenter bees provide locally a high visual acuity (Somanathan et al. 2009), which is used for mate detection (Somanathan et al. 2017). An attractive hypothesis therefore is that the carpenter bee’s bright wing colours play an important role in intraspecific signaling. Here we study the *X. latipes* wing optics and structures that determine the prominent colouration and polarization.

## Materials and methods

### Specimens and photography

Specimens of *X. latipes* were purchased from Bugmaniac.com. Close-up micrographs of small wing areas were made with a Zeiss Universal microscope, using a Zeiss Epiplan 16x/0.35 objective (Zeiss, Oberkochen, Germany).

### Micro-spectrophotometry

Reflectance and transmittance spectra of local wing areas (size 10 × 10 µm^2^) were measured with a microspectrophotometer (MSP), consisting of a Leitz Ortholux microscope with a LUCPlanFL N 20x/0.45 objective (Olympus, Tokyo, Japan) and an Avantes AvaSpec-2048-2 CCD detector array spectrometer (Avantes, Apeldoorn, Netherlands). The reference for the reflectance measurements was a white diffuse standard (Avantes WS-2). The local wing thickness was derived from the periodic oscillations in the wing reflectance spectra with a procedure as outlined previously (Stavenga 2014).

### Electron microscopy

Electron microscopy was performed using standard protocols. Briefly, primary fixation was performed in 2% glutaraldehyde and 2% paraformaldehyde in 0.1 M cacodylate buffer (pH 7.4). Cells were osmicated prior to embedding with 1.5% osmium tetroxide/potassium ferrocyanide. Ultrathin (80 nm) sections were placed on single slot (2 × 1 mm) copper grids and contrasted with neodymium (Kuipers and Giepmans 2020). Acquisition of EM images was performed with a Zeiss Supra55 ATLAS.

#### Multilayer modelling

Assuming that the wing can be treated as a multilayer consisting of different compositions of chitin and melanin, wing reflectance and transmittance spectra were calculated for normally incident light with a matrix transfer procedure (Yeh 2005; Stavenga 2014; Stavenga et al. 2018), using the refractive indices of butterfly chitin (Leertouwer et al. 2011) and melanin (Stavenga et al. 2015).

### Imaging scatterometry

To visualize the far-field angular distribution of the scattered light, imaging scatterometry was applied to wing pieces, glued at the end of pulled glass micropipettes. The sample was positioned in the first focal point of the scatterometer’s ellipsoidal mirror, which collects light from a full hemisphere. A narrow aperture (5º) beam provided by a xenon lamp illuminated a small area of a scale (diameter 13 μm). Scatterogram images were acquired by an Olympus DP70 camera (for details, see Stavenga et al. 2009).

### Angle-dependent reflectance spectra

Wing reflectance spectra were measured as a function of angle of light incidence for both TE- and TM-polarized light in a goniometric setup with two rotatable optical fibres. One fibre (aperture 6°, distance to object 5 cm) delivered light from a xenon lamp to the object, and the other fibre (aperture 6°, distance to object 10 cm), equipped with a linear polarizer, collected the reflected light and guided it to the spectrometer. The angular resolution of the setup has a Gaussian shape with half-width 5º (Stavenga et al. 2011). The reflectance peak wavelength (*λ*_max_) of the measured spectra was interpreted by considering that the reflectance peak wavelength of a multilayer consisting of layers of two media with refractive indices *n*_1_ and *n*_2_ and alternating in thickness *d*_1_ and *d*_2_, when illuminated by a beam with angle of incidence ${\theta }_{0}$ from a medium with refractive index *n*_0_ is given by

1a $${\lambda }_{\text{max}}\left({\theta }_{0}\right)=2({n}_{1}{d}_{1}\text{cos}{\theta }_{1}+{n}_{2}{d}_{2}\text{cos}{\theta }_{2})$$

with

1b $${n}_{0}\text{sin}{\theta }_{0}={n}_{1}\text{sin}{\theta }_{1}={n}_{2}\text{sin}{\theta }_{2}$$

When ${n}_{1}\approx {n}_{2}$, or ${\theta }_{1}\approx {\theta }_{2}$, it follows that

2 $${\lambda }_{\text{max}}\left({\theta }_{0}\right)\approx {\lambda }_{\text{max}}\left(0\right)\sqrt{1-{(\text{sin}{\theta }_{0}/{n}_{1})}^{2}}$$

### Calculating photoreceptor signals

How the wing reflections will be detected by conspecifics can be assessed by assuming a set of three classes of photoreceptors with maximal sensitivity in the ultraviolet (UV), blue (B), and green (G) wavelength ranges. The relative signals created in the three receptor classes, *i* = 1–3, are

3 $${S}_{i,\text{T}}\left(\theta \right)=\int {R}_{\text{T}}\left(\lambda ,\theta \right){V}_{i}\left(\lambda \right)I\left(\lambda \right)d\lambda /\int {V}_{i}\left(\lambda \right)I\left(\lambda \right)d\lambda$$

where ${R}_{\text{T}}\left(\lambda ,\theta \right)$ is the wing reflectance spectrum as a function of wavelength $\lambda$ and angle of light incidence $\theta$ for either T = TE- or TM-polarized light; ${V}_{i}\left(\lambda \right)$ is the absorption spectrum of the visual pigment of receptor class *i*. In the calculations, the peak wavelength of the bee’s UV, B, and G receptors was assumed to be 350, 440, and 530 nm, respectively (van der Kooi et al. 2021), and the absorption spectra were calculated by applying standard formulae (Govardovskii et al. 2000; Stavenga 2010); for $I\left(\lambda \right)$ the normalized solar photon flux was used.

The degree of polarization of the signals created by TE- and TM-polarized light, was calculated with.

4 $${D}_{i,\text{pol}}=({S}_{i,\text{TE}}-{S}_{i,\text{TM}})/({S}_{i,\text{TE}}+{S}_{i,\text{TM}})$$

## Results

### Reflectance spectra depend on the location in the wing

**Fig. 2:**
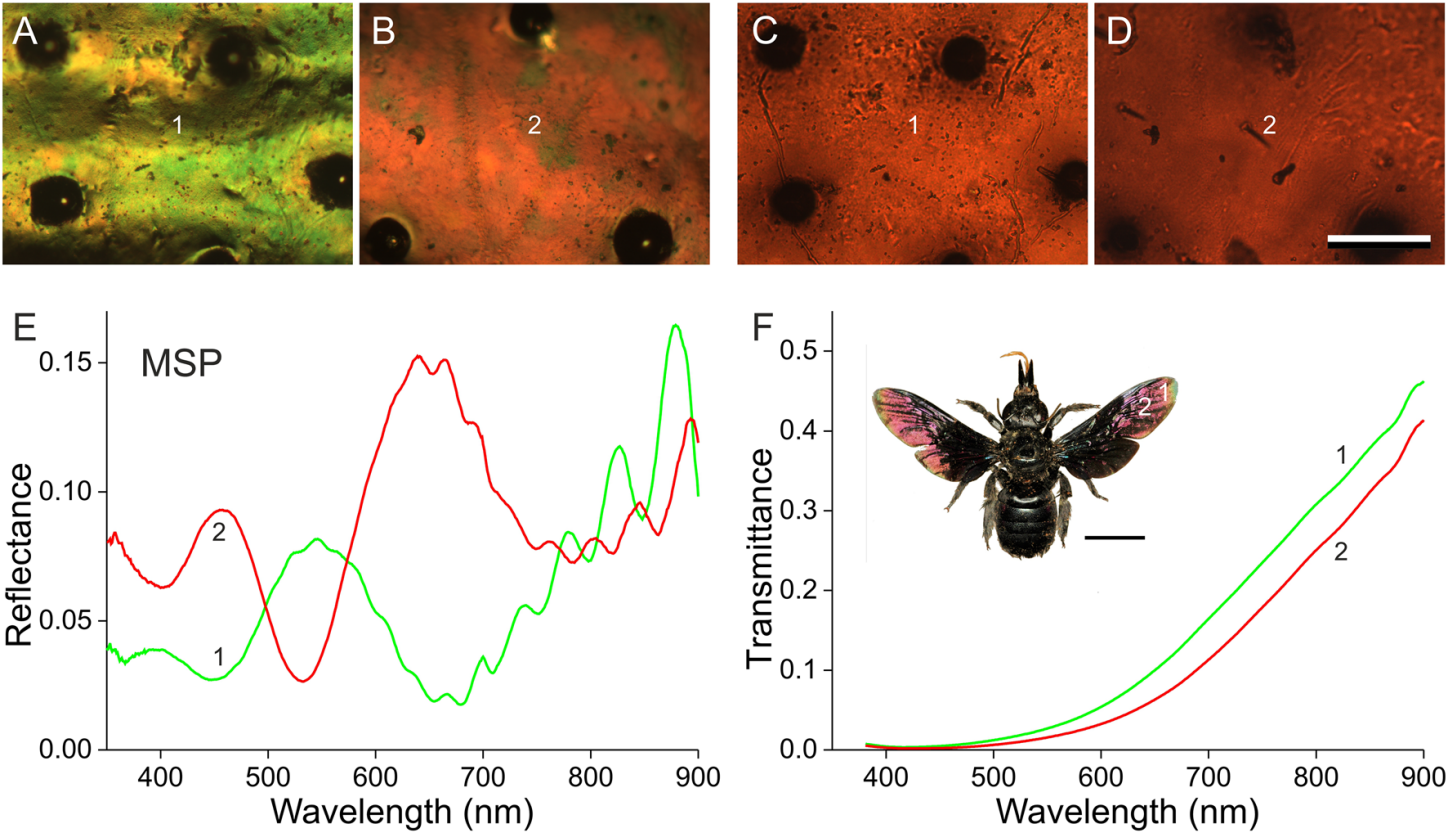
Light microscopy and microspectrophotometry (MSP) of a *X. latipes* wing. **A** Epi-illumination micrograph, 2 mm from the dorsal wing tip (location 1). **B** Epi-illumination micrograph, 7 mm from the wing tip (location 2). **C** Transmitted light micrograph at the same location of panel A. **D** Transmitted light micrograph at the same location of panel B (**A**–**D** scale bar: 100 μm). **E** Reflectance spectra of a 10*10 µm^2^ sized area at 2 and 7 mm from the wing tip (locations 1, and 2, respectively). **F** Transmittance spectra measured at locations 1 and 2 (inset scale bar 1 cm)

The dorsal as well as the ventral sides of the wings of both male and female *X. latipes* display upon epi-illumination a distinct colour pattern that rather varies with the location on the wing (Figs. 1,  2A, B). The correspondingly varying reflectance spectra consist of broad bands in the visible wavelength range together with periodic oscillations in the longer wavelength range (Fig. 2E). The latter oscillations can be readily understood as being due to the total wing acting as a thin film reflector. By applying the analysis described previously (Stavenga 2014), the mean wing thickness at a distance 2 and 7 mm from the wing tip (Fig. 2, #1, 2) was thus estimated to be 4.4 and 5.1 μm, respectively.

In transmitted light, the wings are more or less universally brown-red (Fig. 2C,D). The measured transmittance spectra of the two locations (Fig. 2F) are characteristic for melanin (see e.g. Figure 1F of Stavenga et al. 2012) and show that the concentration of melanin is substantial. The transmittance measurements showed that the melanin density increases with an increasing distance from the wing tip.

**Fig. 3:**
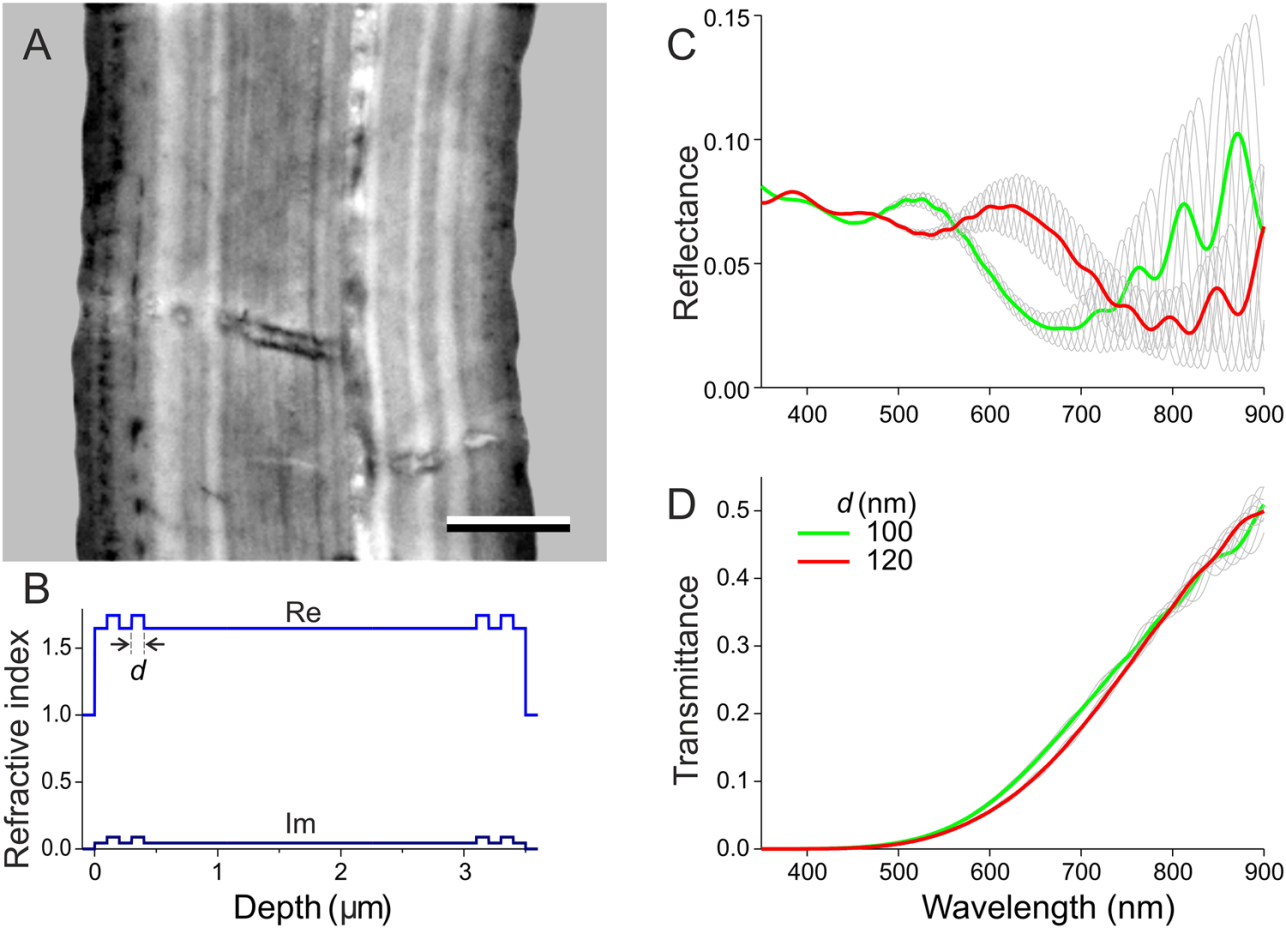
Wing anatomy and optical characteristics. **A** Transmission electron microscope cross section of the wing (scale bar: 1 μm). **B** Model refractive index profile at wavelength 500 nm (Re: real part; Im: imaginary part) due to peripheral layers with thickness *d =* 100 nm. **C** Calculated reflectance spectra resulting from the refractive index profiles as in panel B, with thicknesses *d =* 100 and 120 nm; the green and red curves are the average of the spectra of wings with total thickness 3420, 3460, 3500, 3540, and 3580 nm. **D** Corresponding transmittance spectra

### Thin layers of melanin can explain the varying spectral properties

The images and spectra clearly demonstrate a structural origin of the wing colouration. To investigate the structure of the carpenter bee wing, we performed electron microscopy (Fig. 3A). Cross sections of the wing show that the wing is highly layered. Previous studies on jewel beetle elytra (Stavenga et al. 2011) and odonatan wings (Vukusic et al. 2004; Schultz and Fincke 2009; Stavenga et al. 2012; Guillermo-Ferreira et al. 2015) provided convincing evidence that a higher electron density indicates a higher melanin concentration and that melanin is specifically deposited in a number of regularly arranged, peripheral layers. The carpenter bee’s wing sections did not show very distinct melanin layers, and therefore we created a heuristic model of a wing with overall a 50% chitin, 50% melanin content, while containing in the periphery two equally spaced layers with a pure melanin content. Figure 3B shows the case of a 3500 nm thick wing in air, with near both borders two pure melanin layers with thickness and inter-distance *d* = 100 nm (Fig. 3B). The local refractive index is a weighted sum of the chitin and melanin composition, where the melanin absorption determines the imaginary component of the complex refractive index profile (shown in Fig. 3B for a wavelength of 500 nm).

The reflectance spectrum of the model wing, calculated with a matrix transfer procedure, showed very large amplitude oscillations in the long-wavelength range. These are due to interference of the light reflected on both wing surfaces, which in the modelling are assumed to be perfectly parallel, i.e., the wing thickness is assumed to be constant. As in reality the wing thickness varies, we calculated the reflectance spectra of 5 wings with average thickness 3500 nm, differing in thickness by 40 nm: 3420, 3460, 3500, 3540, and 3580 nm. We considered for each wing thickness two cases, with melanin layer thickness *d* = 100 nm and *d* = 120 nm (Fig. 3C, thin grey curves). Subsequently we calculated for each case the mean spectrum of the five wing thicknesses (Fig. 3C, green and red curves, respectively). Figure 3D shows the transmittance spectra corresponding to the spectra of Fig. 3C.

The calculated reflectance spectra are highly dependent on the wing thickness, especially in the longer wavelength range (Fig. 3C). Modelling furthermore shows that the shape of the reflectance spectra in the shorter wavelength range strongly differs between the two cases where the melanin layers differ in thickness (Fig. 3C). However, concerning the transmittance spectra, the modelling shows that they are virtually fully determined by the wing’s total melanin content (Fig. 3D). An important note here is that only the peripheral layers at the side of the incident light determine the reflectance, because the high melanin absorption strongly reduces the light flux in the shorter wavelength range before it reaches the layers on the opposite side, so that the layers at the other side play a negligible role.

**Fig. 4:**
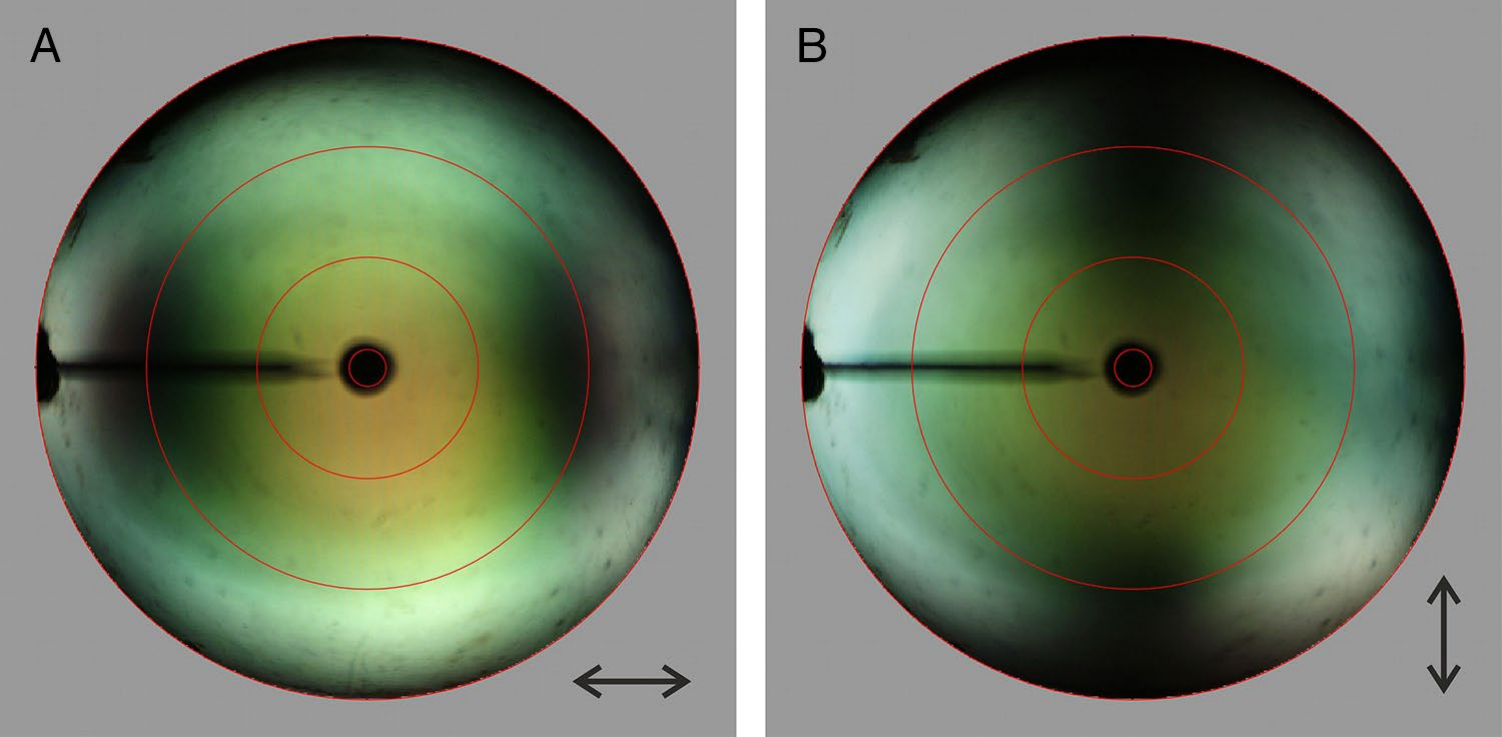
Imaging scatterometry of a *X. latipes* wing with hemispherical illumination. **A** Scatterogram for horizontally polarized light. **B** Scatterogram for vertically polarized light. The red circles indicate scattering angles of 5, 30, 60, and 90°

### The wing reflections become highly polarized at large angles of light incidence

The carpenter bee wing’s structural colouration is highly iridescent, and we therefore investigated this by performing imaging scatterometry. Applying a broad-band (white) hemispherical illumination with either horizontally (Fig. 4A) or vertically (Fig. 4B) polarized light yielded similar scatterograms, except for the 90° rotation. The two scatterograms, which slightly differ due to the venation and irregularities in the wing structure (Figs. 1, 2A–D), display an orange–red center, which corresponds to the reflectance spectra measured in about normal directions (Fig. 2E). With an increasing angle of reflection, the colour initially shifts to green, but at large reflection angles, the colour gradually changes into a broad-band white. Notably, at Brewster’s angle (~ 50–60°), where the TM-polarized light is extinct, the reflection intensity is low (Fig. 4A, B).

**Fig. 5:**
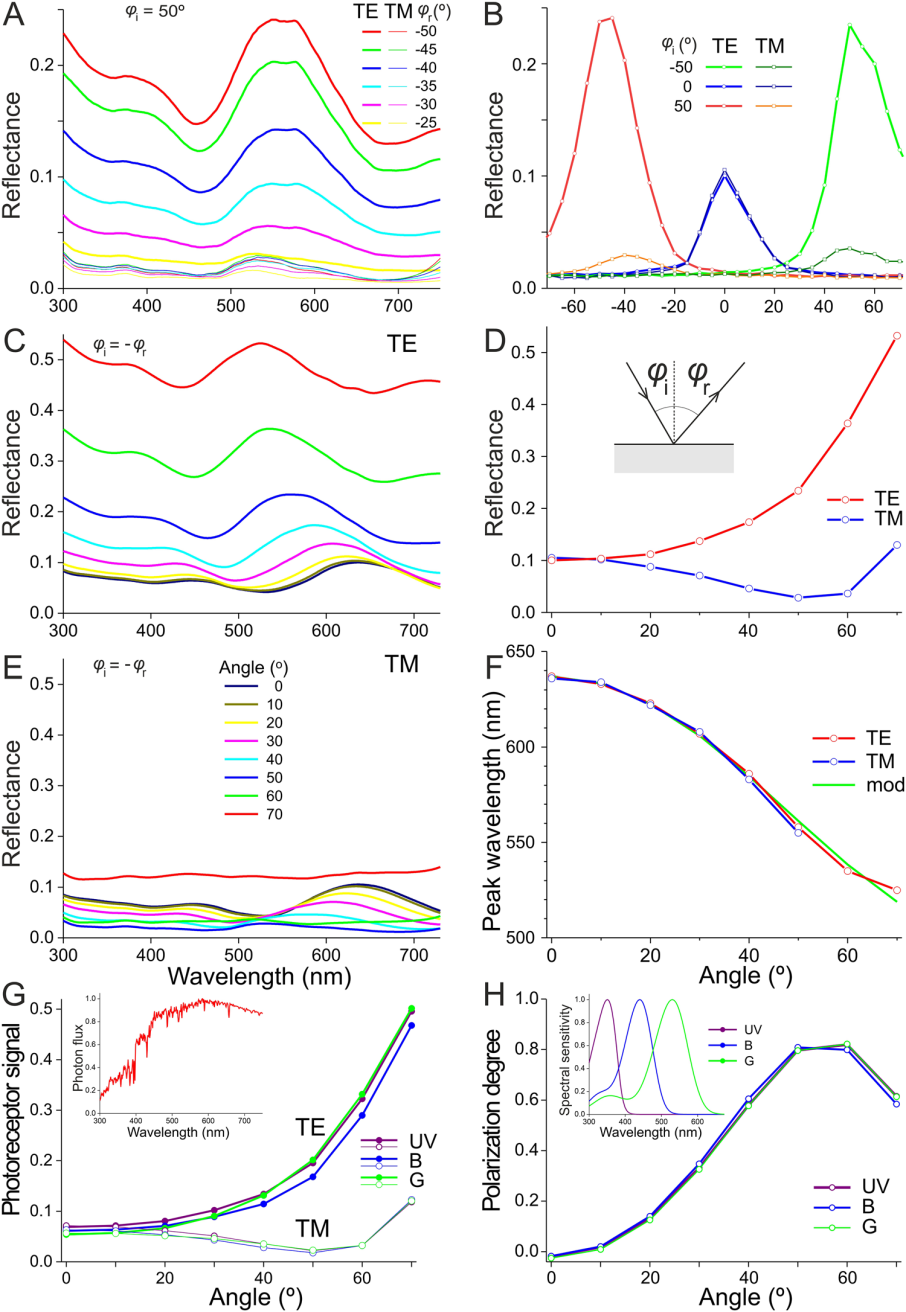
Angle-dependent reflectance measurements of a *X. latipes* wing. **A** Angular spread measured with a fixed angle of light incidence *φ*_i_ = 50° and a variable angle of reflection *φ*_r_ = 50, 45,…, – 25° (see inset panel D), for TE- and TM-polarized light. **B** Peak reflectance for fixed angles of light incidence, *φ*_i_ = – 50, 0, and 50° as a function of the angle of reflection, *φ*_r_. **C** Specular reflectance spectra for *φ*_i_ = – *φ*_r_ = 0°–70° for TE-polarized light. **D** Peak specular reflectance (*φ*_i_ = – *φ*_r_) for TE- and TM-polarized light derived from panels C and E. **E** Specular reflectance spectra for *φ*_i_ = – *φ*_r_ = 0°­–70° for TM-polarized light. **F** Peak wavelength of the specular reflectance spectra as a function of the angle of light incidence (*φ*_i_ = – *φ*_r_), for TE- and TM-polarized light, together with a model fit (mod). **G** Angle dependence of the TE- and TM-signal, elicited by sunlight (inset) reflected by the wing, in a UV-, B-, and G-photoreceptor, with absorption spectrum as in inset panel H. **H** Polarization degree of the sunlight reflected by the wing and absorbed by the three receptor types as a function of the angle of light incidence

We investigated this into more detail by measuring the reflectance as a function of the angle of illumination with a setup consisting of two rotatable fibres. The size of the illuminated wing area was in the order of several mm, but the wing thickness and thus the reflectance spectrum locally vary. The measured reflectance spectra will therefore be the integral of (slightly) varying spectra. Furthermore, the wing surface is not an ideal flat plane, and the unevenness will hence cause an angular spread of the reflected light. This was first tested for an angle of illumination *φ*_i_ = 50° (near Brewster’s angle) by measuring the reflectance spectra as a function of the angle of reflection around the mirror angle, *φ*_r_ = – 50°; see inset Fig. 5D. For both TE- and TM-polarized light, the spectra obtained at different reflection angles appeared to be about proportional (Fig. 5A), but the amplitude of the reflectance spectra measured for TE-polarized light (bold lines) was much larger than the reflectance amplitude for TM-polarized light (thin lines, Fig. 5A, B). The half-width of the angular spread of the reflectance amplitude was ~ 30° (Fig. 5B). When the angle of light incidence was *φ*_i_ = 0° and *φ*_i_ = – 50°, the half-width of the angular spread of the reflectance amplitude was also ~ 30° (Fig. 5B).

We therefore measured subsequently the reflectance as a function of the angle of light incidence only at the mirror angle (*φ*_i_ = – *φ*_r_). The reflectance spectra thus obtained for TE- and TM-polarized incident light were strongly angle-dependent. With an increasing angle of light incidence, the reflectance amplitude increases monotonically for TE-polarized light (Fig. 5D), exhibits a valley at Brewster’s angle for TM-polarized light (Fig. 5D), and shows the same hypsochromic shift of the reflectance peak wavelength for both TE- and TM-polarized light (Fig. 5F). These characteristics strongly indicate the presence of a dielectric multilayer (Fig. 5C,E). The peak wavelength is indeed well approximated by Eq. 2 using a refractive index *n*_1_ = 1.62, which is about the mean of the refractive indices of chitin and melanin around 600 nm (Fig. 5F).

The wing reflections may serve as a signal for conspecifics. We therefore assume that sunlight (inset Fig. 5G) is reflected by the wings and is observed by a trichromatic visual system, equipped with three photoreceptor classes, UV, B, and G (inset Fig. 5H). The signals detected by the three photoreceptors as a function of the angle of light incidence at the mirror angle, calculated for TE-polarized light using Eq. 2, appear to be virtually the same for all receptor types (Fig. 5G, solid symbols), and the same holds for TM-polarized light (Fig. 5G, open symbols). Accordingly, the angle-dependence of the degree of polarization is also the same (Fig. 5H). The degree of polarization reaches a value of ~ 0.8 at Brewster’s angle.

## Discussion

Many species of carpenter bees (*Xylocopa*) are brightly coloured due to metallic-reflecting bodies and wings, which is caused by multilayers of melanin in a chitin–melanin matrix. At about normal illumination, the multilayer reflectance is low (~ 0.1), even at the peak wavelength, but the reflectance increases with skew illuminations. The overall high melanin content causes a very effective absorbing background, so that a distinctly coloured wing pattern arises. Very similar structural colouration phenomena can be seen in the bodies and wings of numerous insects (Vukusic et al. 2004; Schultz and Fincke 2009; Stavenga et al. 2012; Guillermo-Ferreira et al. 2015).

The displayed strong iridescences are accompanied by considerably polarized reflection patterns, phenomena widespread among arthropods (Cronin et al. 2014). The distinct colour and polarization signals may possibly function in intraspecific recognition. Whether that holds for carpenter bees is an attractive hypothesis, as these bees possess acute vision that has been demonstrated to be used in mate recognition (Somanathan et al. 2017). However, in honey bees, polarization vision appears to be restricted to the dorsal area of the compound eyes (e.g. Menzel and Snyder 1974). In the case of polarization-insensitive photoreceptors, the angle-dependent sensitivity is due to the average of the photoreceptor signals for TE- and TM-polarized light (Fig. 5G). The signal will then only substantially increase at an angle of illumination above ~ 50°. The angle-dependent reflection amplitude may act as an important visual signal.

The role of the body colouration is far from well understood, but indirect evidence can be derived from the case of *Xylocopa iris*. The reflecting wings of the females when at rest display a distinct pattern that mimics the orchids *Ophrys spuneri* and *O. sipontensis* (Spaethe et al. 2007). Males attracted by this pattern land sequentially on different orchids and thus fulfil a pollinating function. Other carpenter bees do not have strongly iridescent wings but are rather uniquely coloured by a cover of pigmented hairs on thorax and abdomen, as e.g. *X. caerulea* and *X. confusa*, a topic that deserves a separate treatise.

## Data Availability

This article has no additional data.
